# Impact of a Simulation-Based Communication Workshop on Resident Preparedness for End-of-Life Communication in the Intensive Care Unit

**DOI:** 10.1155/2015/534879

**Published:** 2015-06-25

**Authors:** Abraham Markin, Diego F. Cabrera-Fernandez, Rebecca M. Bajoka, Samantha M. Noll, Sean M. Drake, Rana L. Awdish, Dana S. Buick, Maria S. Kokas, Kristen A. Chasteen, Michael P. Mendez

**Affiliations:** Department of Medicine, Henry Ford Hospital, 2799 West Grand Boulevard, Detroit, MI 48202, USA

## Abstract

*Introduction*. Although residents frequently lead end-of-life (EOL) discussions in the intensive care unit (ICU), training in EOL care during residency has been required only recently, and few educational interventions target EOL communication in the ICU. This study evaluated a simulation-based intervention designed to improve resident EOL communication skills with families in the ICU. *Methods*. Thirty-four second-year internal medicine residents at a large urban teaching hospital participated in small group sessions with faculty trained in the “VitalTalk” method. A Likert-type scale questionnaire measured self-assessed preparedness before, immediately following, and approximately 9 months after intervention. Data were analyzed using Wilcoxon rank-sum analysis. *Results*. Self-assessed preparedness significantly improved for all categories surveyed (preintervention mean; postintervention mean; *p* value), including discussing bad news (3.3; 4.2; *p* < 0.01), conducting a family conference (3.1; 4.1; *p* < 0.01), discussing treatment options (3.2; 3.9; *p* < 0.01), discussing discontinuing ICU treatments (2.9; 3.5; *p* < 0.01), and expressing empathy (3.9; 4.5; *p* < 0.01). Improvement persisted at follow-up for all items except “expressing empathy.” Residents rated the educational quality highly. *Conclusion*. This study provides evidence that brief simulation-based interventions can produce lasting improvements in residents' confidence to discuss EOL care with family members of patients in the ICU.

## 1. Introduction

Conducting end-of-life (EOL) discussions with patients' families is an essential skill for residents in the intensive care unit (ICU). Approximately 22% of all US deaths occur in the ICU [1], and 75% of patient care decisions in the ICU are made by family members [2]. Effective physician-patient communication has been shown to improve measurable outcomes [3], including decreased ICU length of stay [4, 5] and reduced psychological distress among patients' families [6, 7]. EOL discussions have been associated with less aggressive care near death and earlier hospice referrals [8]. Accordingly, effective communication in the ICU has been embedded in national quality measures [9].

Although the Accreditation Council for Graduate Medical Education has mandated training in EOL care for internal medicine residents [10], a nationally standardized curriculum does not exist. Research has shown suboptimal quality of EOL discussions [11, 12], and many residents remain uncomfortable discussing EOL care [13]. Simulation-based educational interventions have been shown to improve residents' ability to use recommended EOL communication skills [14–16], but few have focused on improving resident communication skills for family meetings in the ICU setting. In addition, many published educational interventions for residents were studied before EOL education was required in medical school [17], leaving their effectiveness for current residents unknown.

This study used a pretest-posttest design to evaluate the effectiveness of a brief simulation-based training program intended to improve residents' EOL discussions with families in the ICU. The course was modeled on prior simulation-based interventions [16, 18, 19].

## 2. Methods

### 2.1. Participants

All of the 35 residents in internal medicine at our academic medical center preparing to start postgraduate year 2 in June 2013 were eligible to participate in the study. The EOL educational intervention was a required educational activity for eligible participants and implemented along with standard clinical rotations. This study was deemed exempt from informed consent per the health system's institutional review board.

### 2.2. Educational Intervention

Two faculty members attended two 3-day workshops (VitalTalk, http://www.VitalTalk.org) to learn to facilitate small-group ICU communication skills sessions using simulated patient family members. Residents participated in three 90-minute sessions in groups of 5 to 7 under the guidance of a primary faculty facilitator and a cofacilitator. Simulations were repeated until all eligible residents participated.

The educational sessions focused on discussing serious news with families in the ICU, using the core skills of “ask-tell-ask,” responding to family emotion with empathy as guided by the “NURSE” mnemonic (naming, understanding, respecting, supporting, and exploring), and transitioning goals of care [20]. The educational format included (1) a written module sent 1 week prior to the session, (2) a short didactic overview of the core skills, (3) faculty demonstration, and (4) resident practice with a simulated ICU family member. Residents took turns practicing with a simulated family member, who was an improvisational actor trained to respond differently depending on the effectiveness of the resident's communication skills. The rest of the group and the facilitators provided constructive feedback on the resident's communication skills and helped to brainstorm alternative communication strategies in areas of conversation where the resident felt stuck. The resident was then able to immediately implement the feedback by “rewinding” the simulated-encounter to the “stuck-point” and use the new communication skill.

### 2.3. Measurement and Outcomes

A survey on attitudes toward EOL care was administered prior to the initial small group session for baseline measurement, immediately following the educational intervention, and approximately 9 months later. The survey contained 6 questions measuring self-assessed preparedness to deliver various aspects of EOL care using a 5-point Likert scale. Data on demographic information, future career plans, prior structured educational experiences, and prior bedside teaching relevant to EOL communication skills were also collected. A question regarding previous formal training in discussing a clinical trial with critically ill patients in the ICU, to which few residents were expected to have been exposed, served as a measure of internal validity.

The primary outcome of the study was change in resident self-assessed preparedness to discuss EOL care with patients' families. Secondary outcomes included assessment of the structured and bedside EOL education received by residents during medical school and the first year of residency, as well as satisfaction with the course.

### 2.4. Data Analysis

Descriptive statistics were used to analyze demographic information. The Friedman test and Wilcoxon rank-sum tests were used to compare results of the assessments. All analyses were performed using SAS version 9.4 (SAS Institute Inc., Cary, NC). Data were deidentified after completion of all analyses; data could not be immediately deidentified because subject identifiers were required for pairing pretest and follow-up data points. No identifiable participant data were shared with residents' supervisors.

## 3. Results

### 3.1. Demographics

Of the 35 internal medicine residents eligible for the study, 34 attended the workshop. One was not able to attend due to scheduling conflict. A total of 33 completed both the presurvey and immediate postintervention survey and 15 residents completed the 9-month follow-up survey.  Table 1 shows demographic information of the participants.

### 3.2. Prior Formal Education and Experience

Most residents reported previous structured teaching on discussing code status (88%), expressing empathy (88%), and giving bad news to a family (82%). Less structured teaching was reported for discussing discontinuing intensive care treatments (36%) or discussing religion or spiritual issues with patients and families (24%). Few reported discussing a clinical trial for a patient in the ICU (3%). The same pattern prevailed for previous bedside teaching (Table 2). Most residents (65.6%) had already led a family meeting prior to the intervention.

### 3.3. Self-Assessed Preparedness for EOL Communication Tasks

**Figure 1:**
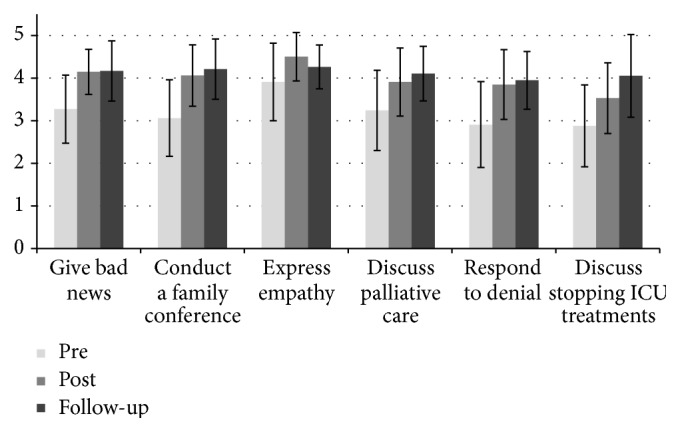
Resident self-assessed preparedness prior to the intervention (*n* = 38), after the intervention (*n* = 32), and at 9-month follow-up (*n* = 18). All *p* < 0.05 except “expressing empathy” at follow-up (*p* = 0.12). Error bars show standard deviation. ICU, intensive care unit.

Self-assessed preparedness significantly increased immediately following the intervention for all items surveyed (Figure 1). Mean score increases on the 5-point Likert scale (1, not well prepared; 3, somewhat prepared; 5, very well prepared; with 2 and 4 corresponding to intermediate values) were significant: giving bad news to a family (0.91, *p* < 0.01), conducting a family conference (1.0, *p* < 0.01), expressing empathy (0.58, *p* < 0.01), discussing treatment options and palliative care with families of critically ill patients (0.66, *p* = 0.01), responding to families who deny the seriousness of their loved one's illness (0.94, *p* < 0.01), and discussing discontinuing intensive care treatments (0.67, *p* < 0.01). The Friedman test revealed significant differences between the 3 assessments. Wilcoxon signed-rank tests demonstrated that scores improved to initial baseline for all items at the 9-month follow-up (Figure 1) except for “expressing empathy” (*p* = 0.12).

### 3.4. Workshop Evaluation by the Participants

**Figure 2:**
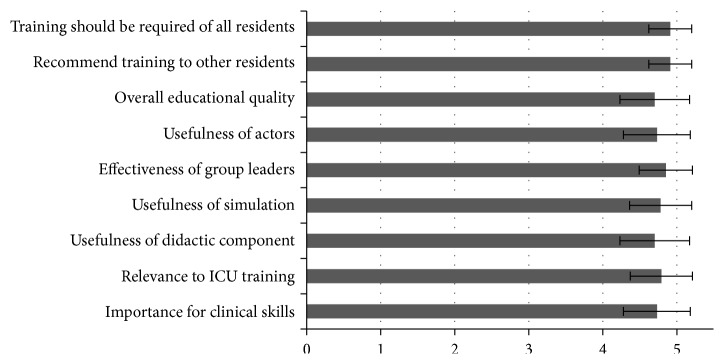
Resident satisfaction with intervention (*n* = 32; 1, Poor; 2, Fair; 3, Good; 4, Very Good; 5, Excellent). Error bars show standard deviation. ICU, intensive care unit.

The intervention was favorably evaluated by the residents, including the utility of the interactive didactics, use of actors for skill practice, and readability and utility of the presession modules (Figure 2). Additionally, more than 90% of residents “strongly agreed” that this training should be required for all internal medicine residents.

## 4. Discussion

This study showed that resident self-assessed preparedness for EOL communication with families in the ICU significantly improved after a brief didactic and simulation-based intervention. Scores remained significantly higher at 9-month follow-up compared to the preintervention baseline, and the course was evaluated favorably by residents. These results were observed in a sample of residents who reported considerable previous training in EOL communication during medical school and their intern year.

Our findings are concordant with previous work using a similar curriculum with critical care fellows in the ICU [19] and non-ICU work demonstrating that brief interventions can significantly improve trainees' EOL communication skills [14, 21, 22]. Unlike previous studies, ours focused specifically on improving resident communication skills with families in the ICU and also demonstrated improvement nearly 1 year after the intervention.

Lorin et al. reported improvement in objective evaluations of EOL discussion skills among medical students on their ICU clerkship following a brief intervention consisting of a lecture, discussions, and interaction with a simulated patient [23]. Han et al. reported improvement in residents' self-rated communication skills after a supervised clinical interaction with a real patient [21]. In reporting results of a retreat for internal medicine residents, Yuen et al. showed that self-assessed comfort significantly improved immediately following a slide presentation and 4-hour interactive session [24]. Smith et al. integrated a brief teaching session into an internal medicine resident program and showed improvement in resident confidence [25]. However, not all studies demonstrated improvement in self-reported confidence in communication skills [26, 27], and data are sparse regarding the durability of these changes in self-assessed preparedness.

We utilized the VitalTalk curriculum for its distinction from other interventions in the use of simulated family members who were not limited to scripted responses, the group setting with real-time peer feedback, and the ability to “rewind” and “replay” segments of the interaction based on feedback. These unique features may have contributed to its observed efficacy in our study sample, although the relative value of each of these attributes merits further investigation.

Additional strengths of the VitalTalk program include its “train-the-trainer” model that leverages the skills of national experts to improve the care provided by a large number of trainees, its standardization, and its availability to faculty across the country. Of note, the intervention required only 90 minutes of residents' time. Previous work has shown that time away from clinical responsibilities is a significant impediment to education focused on communication skills [28].

While these results are encouraging, they must be interpreted in light of several limitations. No objective measures of communication quality were available for analysis, and self-reported outcomes have been shown to correlate poorly with patient- or family-reported outcomes [29]. Since all eligible residents participated in the educational intervention as part of a program to improve care at our institution, this study is also limited by a lack of a control group. Observed improvement from baseline to 9-month follow-up might be partly accounted for by learning in the course of usual training. This is a single-center study limited by small sample size and by uncertain generalizability to residents in other specialties or geographic locations, although the present sample includes a culturally diverse population of residents.

The present findings have implications for current practice. Though training in EOL care is currently mandated by the Accreditation Council for Graduate Medical Education, the methods currently employed to satisfy this requirement remain inadequate and heterogeneous. Standardization of these curricula might more reliably improve patient care than the current system. That empathy alone did not significantly increase from baseline to follow-up also has implications for graduate medical education. It is possible that immediate improvements in self-assessed empathy were offset over time by the well-documented “empathy decline” among residents during their training [30].

Moving forward, several unanswered questions remain regarding the optimal strategy to improve EOL care provided by residents. Future work may involve comparison of VitalTalk with other published interventions as well as assessing patient and family satisfaction along with objective measures of quality care, such as ICU length of stay and timing of enrollment in hospice. Additionally, strategies specific to the ICU should be integrated into broader systems designed to improve EOL care across the continuum of medical acuity, including outpatient clinics, the emergency department, and non-ICU inpatient units.

## 5. Conclusion

Implementation of the VitalTalk program at a large urban teaching hospital significantly improved resident self-assessed preparedness for EOL communication with family members in an ICU setting. Interventions that improve EOL care are possible without unduly burdensome time commitments and can be perceived as valuable by residents.

## Figures and Tables

**Table 1:** Demographics (*n* = 33).

	Characteristic	Number (%)
Age	20–25	5 (15.2)
	26–30	26 (78.8)
	31–35	2 (6.1)

Gender	Male	22 (66.7)
	Female	11 (33.3)

Graduate type	US graduate	13 (39.4)
	FMG	20 (60.6)

Ethnicity	Caucasian	9 (27.3)
	African American	2 (6.1)
	Asian	4 (12.1)
	East Indian/Pak	8 (24.2)
	Hispanic/Latino	5 (15.2)
	Mixed	1 (3.0)
	Other	4 (12.1)

Religious background	Protestant	3 (9.1)
	Catholic	9 (27.3)
	Muslim	8 (24.2)
	Hindu	6 (18.2)
	Other	7 (21.2)

FMG, foreign medical graduate; Pak, Pakistani.

**Table 2:** Prior educational experiences.

	Structured teaching number (%)	Bedside teaching number (%)
Giving bad news to a family about their loved one's illness	27 (81.8)	22 (66.7)

Conducting a family conference	18 (54.6)	20 (60.6)

Discussing various treatment options, including palliative care, with families of critically ill patients	20 (60.6)	18 (56.3)

Responding to families who deny the seriousness of their loved ones illness	15 (45.4)	14 (42.4)

Discussing discontinuing intensive care treatments	12 (36.4)	7 (21.2)

Talking to family members who want treatments that you believe are not indicated	17 (51.5)	17 (51.5)

Discussing code status	29 (87.9)	27 (84.4)

Discussing religious or spiritual issues with patients and families	8 (24.2)	9 (28.1)

Expressing empathy	29 (87.9)	26 (81.3)

Discussing a clinical trial for a patient in the ICU	1 (3.0)	2 (6.3)

ICU, intensive care unit.
